# Bagging Treatment Influences Production of C_6_ Aldehydes and Biosynthesis-Related Gene Expression in Peach Fruit Skin

**DOI:** 10.3390/molecules190913461

**Published:** 2014-08-29

**Authors:** Ji-Yuan Shen, Lei Wu, Hong-Ru Liu, Bo Zhang, Xue-Ren Yin, Yi-Qiang Ge, Kun-Song Chen

**Affiliations:** 1 Laboratory of Fruit Quality Biology, The State Agriculture Ministry Laboratory of Horticultural Plant Growth, Development and Quality Improvement, Zhejiang University, Zijingang Campus, Hangzhou 310058, China; E-Mails: sjy_sd@zju.edu.cn (J.-Y.S.); wulei.bj@163.com (L.W.); hear2008dream@163.com (H.-R.L.); xuerenyin@zju.edu.cn (X.-R.Y.); akun@zju.edu.cn (K.-S.C.); 2 Physiological Laboratory for South China Fruits, College of Horticulture, South China Agricultural University, Guangzhou 510642, China; 3 China Rural Technology Development Center, Beijing 100045, China; E-Mail: 68511009@163.com

**Keywords:** bagging, C_6_ aldehydes, HPL, LOX, peach fruit

## Abstract

Bagging is a useful method to improve fruit quality by altering its exposure to light, whereas its effect on fruit volatiles production is inconsistent, and the genes responsible for the observed changes remain unknown. In the present study, single-layer yellow paper bags were used to study the effects of bagging treatment on the formation of C_6_ aldehydes in peach fruit (*Prunus persica* L. Batsch, cv. Yulu) over two succeeding seasons. Higher concentrations of *n*-hexanal and (*E*)-2-hexenal, which are characteristic aroma volatiles of peach fruit, were induced by bagging treatment. After bagging treatment, peach fruit had significantly higher LOX and HPL enzyme activities, accompanying increased contents of C_6_ aldehydes. The gene expression data obtained through real-time PCR showed that no consistent significant differences in transcript levels of LOX genes were observed over the two seasons, but significantly up-regulated expression was found for *PpHPL1* after bagging treatment In addition, bagging-treated fruit produced more (*E*)-2-hexenal and had higher expression levels of *PpHPL1* during postharvest ripening at room temperature. The regulatory role of the LOX-HPL pathway on the biosynthesis of *n*-hexanal and (*E*)-2-hexenal in response to bagging treatment during peach fruit development is discussed in the text.

## 1. Introduction

Volatile compounds interacting with sugars and acids contribute predominantly to fruit flavor. In peach fruit, more than 100 volatile compounds have been identified [1], and green-note sensory volatiles, such as *n*-hexanal and (*E*)-2-hexenal, contribute to the formation of the characteristic peach aroma [2,3]. The biosynthesis of volatile compounds is influenced by peach fruit genetics [4], developmental stage [5], and environmental factors, such as temperature [6,7], oxygen [8,9], and light [10,11]. 

As one of the most popular cultivation approaches, fruit bagging is extensively practiced in China and Japan to improve the fruit quality by altering its exposure to light. For example, fruit bagging has been selected as a useful method for the study of anthocyanin synthesis in fruits such as apple [12] and pear [13]. However, there is little information on the effects of bagging on fruit volatiles, and some reports are even contradictory. In the case of peach, bagging with orange paper caused no effect on the aroma compounds of whole peach fruit at harvest, but significantly increased the (*E*)-2-hexenal contents in the peach skin [10]. Another report showed that *n*-hexanal and 2-hexenal, as well as C_6_-related esters, were significantly lower in peach fruit treated with two-layered paper bags (black inner and brown outer papers) compared with non-bagged fruit [11]. These different results indicate the differential influence of bagging on the contents of C_6_ aldehydes, but the mechanism through which bagging affects the formation of peach fruit volatiles remains unclear. 

Volatile *n*-hexanal and (*E*)-2-hexenal are directly derived from the cleavage of 13-hydroperoxide catalyzed by hydroperoxidelyase (HPL); the 13-hydroperoxide is produced by the metabolism of C_18_ polyunsaturated fatty acids, catalyzed by lipoxygenases (LOXs) [14]. LOX can act on C_18_ fatty acids at either the 9ꞌ or 13ꞌ position to generate two types of oxylipins and is thus classified as 9- and 13-LOX. Accordingly, the HPL enzymes are mainly categorized as 9- and 13-HPL depending on their substrate specificity [15], and a third type, namely 9/13-HPL, which has dual 9- and 13-hydroperoxide specificity, has also been described [16]. The advances in understanding of the roles of LOX and HPL in fruit volatile biosynthesis have been previously reviewed [17]. In our previous studies, we cloned four LOX genes and one HPL gene in peach and analyzed the expression patterns of these genes during postharvest ripening at ambient temperature and after storage at low temperature plus subsequent shelf-life [18,19]. The above described reports provide clues that can be further used in the analysis of gene expression and enzyme activity to better understand the regulatory role of bagging on peach fruit aldehyde biosynthesis. 

In the present work, melting peach fruits were treated with bags over two succeeding seasons. First, the effect of bagging on the concentrations of the green-note *n*-hexanal and (*E*)-2-hexenal was analyzed. Second, the gene expression patterns of LOX and HPL and enzyme activities were studied after bagging treatment. Third, the effects of the bagging treatment on the weight, coloration, esters and lactones at harvest were discussed. Based on these findings, a possible mechanism through which bagging affects volatile synthesis in peach fruit was discussed. 

## 2. Results and Discussion

**Figure 1:**
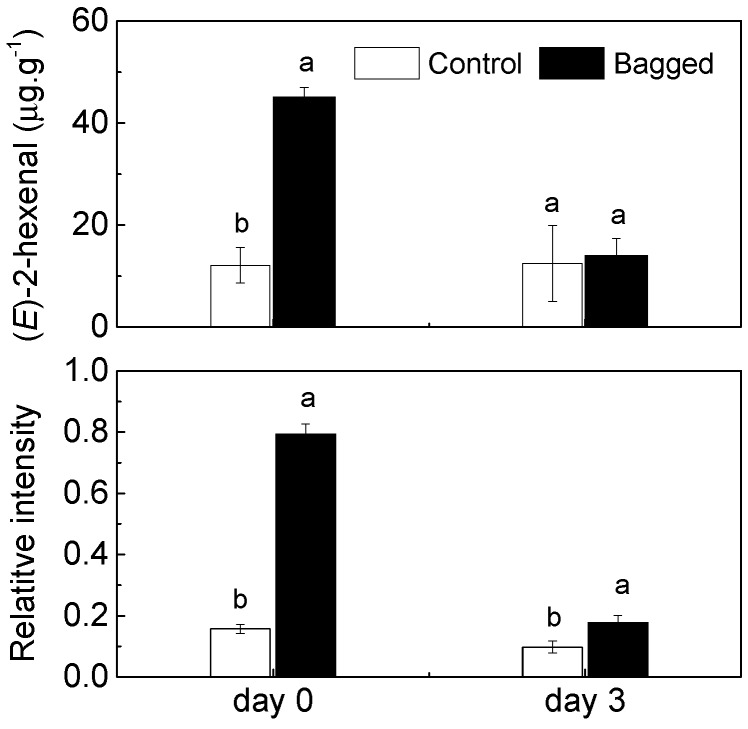
Changes in (*E*)-2-hexenal contents and *PpHPL1* expressions of peach fruit skin during postharvest ripening (2011 fruit season). Each value represents the mean ± standard error of three replicates. The significant differences are indicated with different letters (LSD, 0.05).

Bagging treatment is an effective approach that is used to affect the coloration and flavor of fruits, including the aroma-related volatiles. Of these volatile compounds, C_6_ aldehydes, such as *n*-hexanal and (*E*)-2-hexenal, contribute to the green sensory notes of fruit [3]. Despite the importance of these volatiles, the role of bagging on the regulation of aroma-related volatiles remains unclear. As the characteristic volatile aldehydes for peach flavor, the changes in *n*-hexanal and (*E*)-2-hexenal in peach fruit in response to bagging treatment were analyzed. As shown in Table 1, higher levels of *n*-hexanal were observed in the peach fruits treated with yellow paper bags than in the non-bagged fruits, although differences were not significant. The content of (*E*)-2-hexenal, which is another C_6_ aldehyde, was approximately 60% higher in the bagging-treated fruits than in the controls in the 2010 season, and an accumulation of approximately 3.7-fold was found in the paper bag-treated fruits in the 2011 season (Table 1). When the fruits were held at room temperature for 3 days, contents of (*E*)-2-hexenal declined from 45.09 μg·g^−1^ FW to 14.00 μg·g^−1^ FW in peach fruits treated with bag, while control fruits emitted lower levels during postharvest ripening process (Figure 1). 

**Table 1:** Effects of bagging treatment on the concentrations of *n*-hexanal and (*E*)-2-hexenal (µg·g^−1^ FW) in peach fruit skin at harvest.

	2010 Fruit Season		2011 Fruit Season	
	Control	Bagged	Control	Bagged
*n*-Hexanal	64.03 ± 2.7 ^a^	95.58 ± 11.53 ^a^	26.98 ± 2.46 ^a^	39.49 ± 5.03 ^a^
(*E*)-2-Hexenal	39.98 ± 0.70 ^b^	63.63 ± 2.15 ^a^	12.10 ± 3.48 ^b^	45.09 ± 1.86 ^a^

Note: Each value represents the mean ± standard error of three replicates. Values indicated by different letters are significant different to controls (LSD, 0.05).

Our results showed that the bagging treatment led to remarkably higher accumulations of *n*-hexanal and (*E*)-2-hexenal in peach fruit skin tissue over two succeeding seasons. The induction of C_6_ aldehydes in response to bagging treatment was also reported by Jia *et al.* [10], who observed a significantly higher abundance of (*E*)-2-hexenal in peach fruit skin tissue after bagging with triple parchment paper bags (50% sunlight transmission). In grape fruit, sunlight shading also significantly increased the formation of C_6_ compounds [20]. However, in contrast to the above results, peach fruit bagging with a two-layered paper bag (black inner and brown outer papers) led to a reduction in C_6_ aldehydes [11], but no information on the sunlight transmission of these paper bags was provided. These inconsistencies could be due to the different sunlight transmission properties of the bags used. In addition, fruit cultivars and maturity stages are possibly other important factors that affect volatile compound formation in response to bagging treatment. 

**Figure 2:**
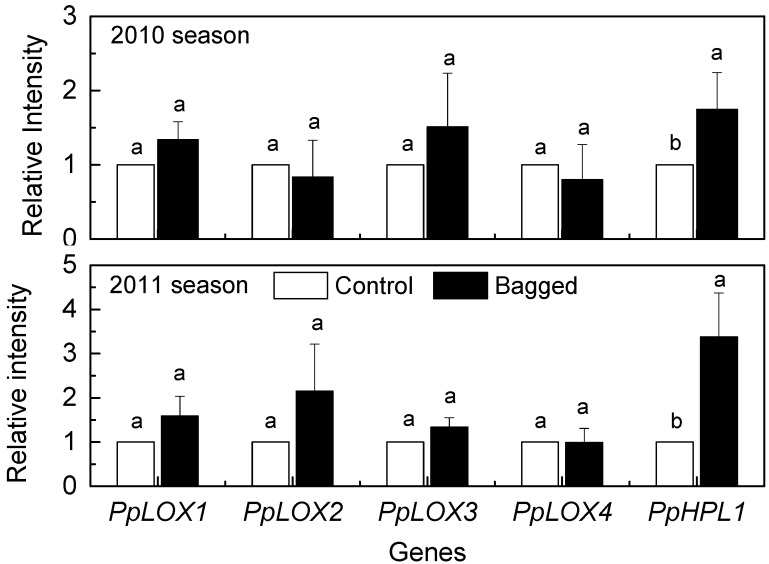
Effect of bagging treatment on the expression of *PpLOXs and PpHPL1* in peach fruit skin. Each value represents the mean ± standard error of three replicates. The significant differences are indicated with different letters (LSD, 0.05).

**Figure 3:**
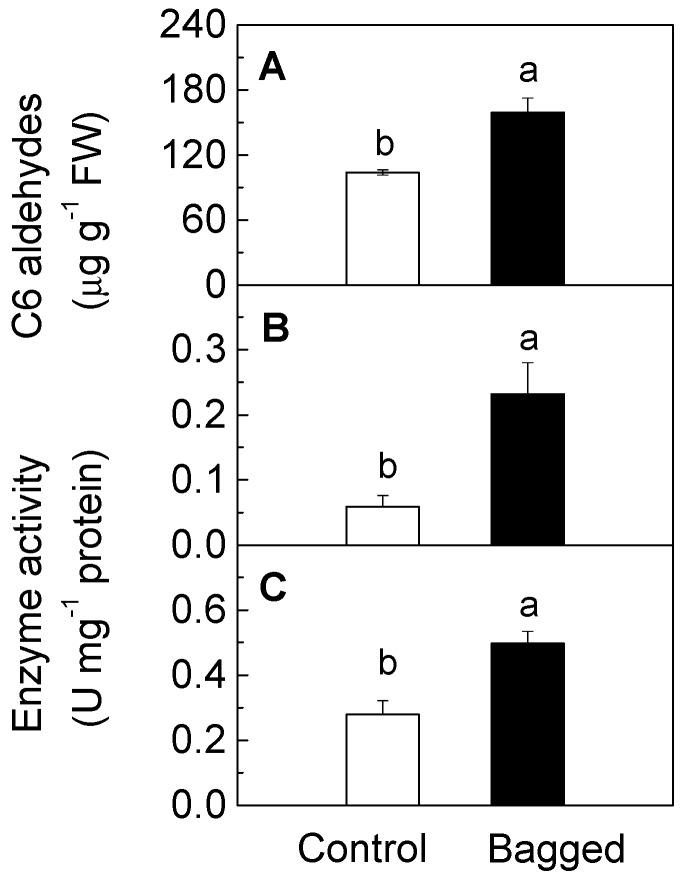
Effect of bagging treatment on the concentrations of C_6_ aldehydes (**A**), LOX activity (**B**), and HPL activity (**C**) in peach fruit skin (2010 fruit season). Each value represents the mean ± standard error of three replicates. The significant differences are indicated with different letters (LSD, 0.05).

The LOX-HPL pathway is considered to be involved in the biosynthesis of C_6_ aldehydes [14]. To clarify the mechanism through which bagging treatment increases the contents of C_6_ aldehydes in peach fruit, the gene expression was analyzed to identify possible genes associated with the observed changes in aldehydes. Transcripts of *PpLOX1* and *PpLOX3* were induced by bagging treatment, however, no significant differences were observed over two succeeding seasons (Figure 2). *PpLOX2* exhibited different expression patterns after bagging, but the levels of difference were both below the 0.05 level of significance. In the case of another member *PpLOX4*, yellow paper bag treatment caused no significant inhibitions in expression levels either in 2010 or 2011 fruit season (Figure 2). Although about four-fold higher LOX enzyme activities were induced after bagging (Figure 3), no significant induction of transcript levels were observed for four LOX genes over two seasons, indicating the existence of other gene family members that contribute to changes in the enzyme activity in response to bagging treatment. 

**Figure A1:**
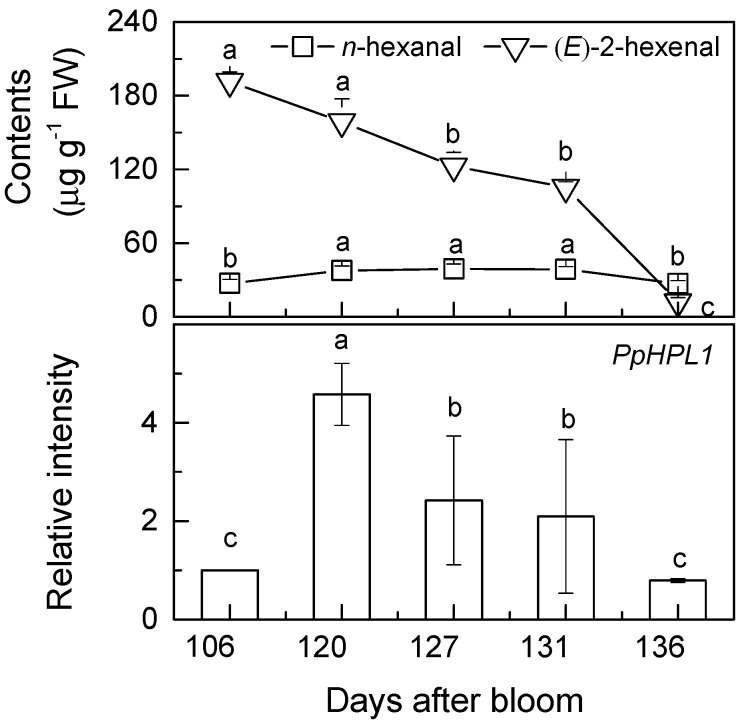
Changes in *n*-hexanal, (*E*)-2-hexenal contents, and *PpHPL1* expression levels of peach fruit skin during maturation. Mean value of three replicates is shown with standard error, and different letters above the symbols indicate significant differences (LSD, 0.05).

With respect to the HPL gene, the bagging treatment significantly induced the accumulation of *PpHPL1* transcripts of peach fruit in the 2010 season, and a significant accumulation of expression levels were found in fruit treated with bags in the 2011 season (Figure 2). Our results also showed that the induction of *PpHPL1* expression was accompanied with an increase in the HPL enzyme activities. Compared with the non-bagged control batch, the bagging treatment induced an approximately 1.8-fold increase in the HPL enzyme activity (Figure 3). In agreement with our previous study [18], expression levels of *PpHPL1* tended to decrease during postharvest ripening at room temperature (Figure 1). High expression levels of *PpHPL1* in bagged peach fruit were approximately 2.3–4.9 times higher than that in the controls (Figure 1). In order to find more evidence to confirm the association of HPL and C_6_ aldehydes, concentrations of the volatile compounds and expression patterns of the gene were analyzed during peach fruit maturation. Significant reduction of (*E*)-2-hexenal contents was observed during the fruit maturation, decreasing from 191.43 μg·g^−1^ at 106 DAB (endocarp harden stage) to 12.10 μg·g^−1^ at 136 DAB (harvest time) (Figure A1). QPCR results showed that transcripts of *PpHPL1* tended to decline as fruit maturing (Figure A1). As expected, expression levels of *PpHPL1* were generally consistent with contents of *n*-hexanal and (*E*)-2-hexenal during the peach fruit maturation. Regression analysis showed that positive correlation R^2^ = 0.83 was observed between aldehydes contents and HPL expression during peach maturation. Our previous study showed that *PpHPL1* belong to 13-HPL group, and was suggested to putatively contribute to the formation of volatiles during postharvest ripening and senescence [18]. Taken together, genes expression dataset indicated that *PpHPL1* was likely associated with the changes in the contents of C_6_ aldehydes in fruit during peach development. 

Formation of volatile compounds is influenced by fruit maturity and ripening stages. Generally, green-note volatiles tended to decrease while contents of fruity-note volatiles accumulated during fruit ripening [17,18,21]. In our present study, the contents of hexyl acetate, (*Z*)-3-hexenyl acetate, and (*E*)-2-hexenyl acetate were slightly lower in the peach fruit treated with bags than that in the controls (Table 2). The bagging-treated peach fruit also produced relatively lower levels of γ-octalactone, γ-decalactone and δ-decalactone (Table 2). Although the levels of esters and lactones were reduced in the bagged fruit, they were generally below the 0.05 level of significance. 

**Table 2:** Effects of bagging treatment on the concentrations of esters and lactones (µg·g^−1^ FW) in peach fruit skin at harvest. These effects were determined in two succeeding seasons.

Volatiles	2010 Fruit Season		2011 Fruit Season	
	Control	Bagged	Control	Bagged
Hexyl acetate	2.28 ± 0.15 ^a^	1.97 ± 0.12 ^a^	0.13 ± 0.06 ^a^	0.06 ± 0.02 ^a^
(*Z*)-3-Hexenyl acetate	2.03 ± 0.26 ^a^	2.02 ± 0.33 ^a^	0.51 ± 0.25 ^a^	0.30 ± 0.03 ^a^
(*E*)-2-Hexenyl acetate	1.47 ± 0.22 ^a^	1.09 ± 0.15 ^a^	0.81 ± 0.21 ^a^	0.68 ± 0.09 ^a^
γ-Octalactone	3.13 ± 0.62 ^a^	2.42 ± 0.36 ^a^	UD	UD
δ-Decalactone	UD	UD	0.37 ± 0.02 ^a^	0.30 ± 0.04 ^a^
γ-Decalactone	0.41 ± 0.09 ^a^	0.32 ± 0.02 ^a^	1.09 ± 0.03 ^a^	0.87 ± 0.11 ^a^

Note: Each value represents the mean ± standard error of three replicates. Values indicated by different letters are significantly different to controls (LSD, 0.05). UD, below the determination limit.

In addition to volatile compounds, differences in peach fruit maturity related quality were also analyzed. The weights of the peach fruits were not significantly affected by bagging treatment in both fruit seasons (Table 3). However, the fruit peel color was significantly changed after bagging treatment. The lightness (*L*) value of the peach fruit treated with yellow bags was significantly higher than that obtained for the control fruits (Table 3). The non-bagged peach fruits had low hue angle (*H*) and chroma (C *) values, and exhibited low contents of chlorophyll a, chlorophyll b, and total chlorophyll (Table 3). Values of fruit firmness were slightly high in bag treated peach fruit, while content of TSS and flesh juiciness were slightly low in fruit treated with yellow bag (Table 3). Although these differences were generally below the 0.05 level of significance, trends were apparent. The above dataset showed that peach fruit after bagging produced higher values of firmness, and had lower juiciness and TSS content, indicating that bagging treatment influence the fruit maturity. 

**Table 3:** Effects of bagging treatment on the weight (g), coloration (*L*, *H_ab_*, *C*), chlorophyll (µg∙g^−1^ FW), firmness, TSS (°Brix) and juiciness (%) of peach fruits at harvest.

	2010 Fruit Season		2011 Fruit Season	
	Control	Bagged	Control	Bagged
Weight	177.23 ± 6.51 ^a^	196.41 ± 3.10 ^a^	134.16 ± 4.34 ^a^	145.38 ± 4.98 ^a^
*L*	57.61 ± 1.06 ^b^	65.76 ± 0.68 ^a^	46.66 ± 0.78 ^b^	59.48 ± 1.22 ^a^
*H_ab_*	79.44 ± 2.22 ^b^	93.71 ± 0.75 ^a^	61.87 ± 0.39 ^b^	94.91 ± 0.68 ^a^
C *	20.52 ± 0.20 ^a^	21.77 ± 0.42 ^a^	20.90 ± 0.65 ^a^	22.26 ± 0.33 ^a^
Chlorophyll a	2.95 ± 0.49 ^a^	2.01 ± 0.35 ^a^	NA	NA
Chlorophyll b	1.77 ± 0.69 ^a^	1.02 ± 0.41 ^a^	NA	NA
Firmness	34.16 ± 1.11 ^a^	34.45 ± 2.69 ^a^	NA	NA
TSS	13.01 ± 0.31 ^a^	12.53 ± 0.45 ^a^	NA	NA
Juiciness	70.02 ± 1.64 ^a^	68.15 ± 2.44 ^a^	NA	NA

Note: Each value represents the mean ± standard error of three replicates. Values indicated by different letters are significantly different to controls (LSD, 0.05). NA means data not available.

Recently, more and more reports have shown that treatment with different light could effectively influence the emissions of plant volatiles [22,23,24], but the molecular basis for this remains unclear. Release of peach draft genome sequence [25] is going to provide an opportunity to clone and identify more genes, thus to understand mechanism of the effects of light on volatiles formation. In addition, it will be interesting to determine whether changes in volatile compounds affected by bagging treatment could affect human perception of flavor quality. 

## 3. Experimental

### 3.1. Plant Materials and Sampling

Melting peach fruits (*Prunus persica* L. Batsch cv. Yulu) were used in this study. The experiments were conducted over two succeeding seasons (2010 and 2011) in the same commercial orchard in Ningbo, Zhejiang Province, China, where bagging fruit with single-layer yellow bag is used as a cultivation method to protect “Yulu” peach fruit. Twenty peach trees were divided into two groups based on the bagging treatment in the 2010 fruit season. Ten fruits of each tree were randomly selected and covered with single-layer yellow paper bags (15.7 cm × 14.7 cm, Zhuangnongyuguo Company, Ningbo, China) on July 19th. The non-bagged peach fruits were exposed to direct sunlight and used as a control. In the 2011 fruit season, nine peach trees of each of ten fruit were covered with single-layer yellow paper bags during the second stage of fruit development (period of slow growth) on 25 July, and fruits from nine other peach plants were not bagged and used as controls. The sunlight transmission analysis using a luminometer (TES-1339, TES Electrical Electronic Corp., Taipei, Taiwan) showed that the sunlight transmission through a single-layer yellow paper bag is about 25%. 

Both the bagged and the non-bagged peach fruits were harvested at commercial maturity (firm-mature stage) over two seasons. To avoid their exposure to light before the quality analysis, the bagged fruits were harvested without removing the bags. The fruits were transported to the laboratory on the day of harvest and screened for uniform size and freedom from defects and wounds. In the second fruit season, peach fruit sampled on 11 July, 25 July, 1 August, 5 August and 10 August, which represent 106, 120, 127, 131 and 136 days after bloom (DAB) respectively. Moreover, fruit harvested at 136 DAB were also allowed for postharvest artificial ripening at room temperature for 3 days. For biochemical and molecular analyses, three replicates of five fruit each were used in each season. 

### 3.2. Fruit Quality Evaluation

The weight and peel color of the fruits were measured on the day of harvest and are shown in Table 1. The fruit peel color, which is expressed through the lightness (*L*), hue angle (*H_ab_*), and chroma (C *), was measured on both sides of the equator of the peach fruit. The lightness was measured directly, and the hue angle and chroma were calculated according to the methods described in a previous study [26]. 

Chlorophyll was extracted from fruit peel as previously described by Lewandowsk *et al.* [27] with some modifications. Fruit peel (200 mg) was ground and homogenized in 90% cold acetone (4 mL) and centrifuged at 10,000 *g* for 20 min at 4 °C. The absorbance of the supernatant was measured using a DU800 spectrophotometer (Beckman, Fullerton, CA, USA) at 644 and 662 nm. The content of chlorophyll a (Chl_a_) and chlorophyll b (Chl_b_) were calculated using following equations: [Chl_a_] = 9.78A_662_ − 0.99A_644_ and [Chl_b_] = 21.40A_644_ − 4.65A_662_. 

A TA-XT2i plus texture analyzer (Stable Micro System, Godalming, England) equipped with a 7.9 mm diameter head was applied for fruit firmness measurement as described by Zhang *et al.* [18]. Two measurements were made on opposite sides of each fruit after the removal of a 1 mm thick skin slice, and data were expressed as newtons (N). 

Total soluble solids (TSS) were measured by slicing both ends of each fruit [18]. Three drops of juice from each slice were then applied to an PR-101α digital hand-held refractometer (Atago, Tokyo, Japan). TSS data were expressed in degrees Brix. 

For juice measurements, 4 flesh cubes were taken from each fruit and the weight of juice released by the pressure produced from a texture analyzer (TA-XT2i Plus) with a 10 cm diameter head was determined. The crushed flesh remaining was also weighed, and the proportion of juice in relation to the total flesh (mass/mass) was calculated and expressed as %, a method previously described by Zhang *et al.* [19]. 

The fruit peel tissue (approximately 1-mm-thick) was sliced using a peeler, frozen in liquid nitrogen, and stored at −80 °C until further use. Three replicates of each of five fruits were used for the volatile and molecular analyses. 

### 3.3. Fruit Volatile Analysis

The volatile compounds in peach fruit were measured using the method described by Zhang *et al.* [18]. Frozen peach peel tissue was ground into a fine powder in liquid nitrogen and one gram was transferred to a 15 mL glass vial containing saturated NaCl solution. Then, 2-octanol (30 µL, Fluka, St. Louis, MO, USA) was added to the blend as an internal standard, and mixture was vortexed for 10 s. The samples were equilibrated for 30 min and subjected to manual solid-phase micro-extraction analysis (SPME) with a fiber coated with 65 µm of polydimethylsiloxane and divinylbenzene (Supelco Co., Bellefonte, PA, USA). Subsequently, the volatile compounds were analyzed on an Agilent 6890N GC equipped with DB-WAX column (0.32 mm, 30 m, 0.25 µm, J&W Scientific, Folsom, CA, USA). The chromatography temperature was programmed as follows: the oven was initially maintained at 34 °C for 2 min, increased to 60 °C at a rate of 2 °C·min^−1^, increased to 220 °C at a rate of 5 °C·min^−1^, and maintained at 220 °C for 2 min. Nitrogen was used as a carrier gas at a flow rate of 1.0 mL∙min^−1^. Using the internal standard value as the reference value, the quantitative values of the volatile compounds were calculated based on the standard curves of the authentic compounds supplied by Sigma-Aldrich (St. Louis, MO, USA). 

### 3.4. Enzymes Activity Analysis

Analysis of LOX activity was performed by the method shown by Echeverria *et al.* [28]. Powdered peel tissue (100 mg) taken from three peaches was homogenized in extraction solution (1 mL). The extraction solution was consisted of 2 mM dithiothreitol, 1 mM EDTA, 0.1% (v/v) Triton X-100, 1% (w/v) PVPP and 0.1 M phosphate (pH 7.5). The substrate solution was consisted of 8.6 mM linoleic acid, 0.25% (v/v) Tween-20, 10 mM NaOH and 0.1 M phosphate (pH 8.0). LOX activity in the crude extract was measured by following spectrophotometrically the increase in absorbance at 234 nm. One activity unit (U) was defined as the increase in one unit of absorbance at 234 nm per minute, and results were expressed as specific activity (U·mg·protein^−1^).

The crude HPL enzyme was extracted according to the method described by Fukushige and Hildebrand [29]. The extraction buffer consisted of 100 mM Tris HCl (pH 8.5), 3 mM EDTA, 3 mM dithiothreitol, 0.5% (w/v) Triton X-100, 1% (w/v) protease inhibitor cocktail, and 5 g·L^−1^ PVPP. One gram of peel tissue was ground and added into 2 mL of extraction buffer, and the homogenate was centrifuged at 12,000 rpm at 4 °C. The enzyme reaction occurred in the presence of NADH and yeast alcohol dehydrogenase, where the aldehyde was converted to alcohol by consuming NADH. The reduction of NADH was spectrophotometrically measured by the loss absorbance at 340 nm. The protein content was measured [30], and the HPL enzyme activity was expressed as U·mg·protein^−1^.

### 3.5. RNA Extraction and Gene Expression Analysis 

The total RNA was extracted from one gram of frozen peel tissue according to the method described by Zhang *et al.* [31]. RNase-free DNase I (Fermentas, St. Leon-Rot, Germany) was used to remove any possible genomic DNA. The first-strand cDNA synthesized from 3 µg of DNA-free RNA was subsequently used for the gene expression analysis of *PpLOX*s and *PpHPL1* using a CFX96 real-time PCR instrument (Bio-Rad, Hercules, CA, USA). The primer sequences of all of the genes assayed and the temperature program for the real-time PCR assay are shown in our previous study [18]. At least three different RNA isolations and cDNA syntheses were used as replicates for the gene expression analysis. The expression levels were expressed as a ratio relative to the levels found in non-bagged fruit, which were set to 1. For peach sampled at differential ripening stages, the first time point (106 DAB) expressions were set to 1. 

### 3.6. Statistical Analysis

The experiment was designed through a completely randomized design. Data from each treatment and control were subjected to analysis of variance (significance at 0.05) and means were separated using least significant difference (LSD). The significant differences are indicated in the figures and tables with different letters. The statistical analysis and figures were generated using OriginPro 9.0 (OriginLab Corporation., Northampton, MA, USA). 

## 4. Conclusions 

In summary, bagging treatment of peach fruit with single-layer yellow paper bags produced more contents of *n*-hexanal and (*E*)-2-hexenal in skin tissues via inducing *PpHPL1* expression and enzyme activity. Our results indicate that covering the fruits with different bags with various sunlight transmission properties is a potential effective method for the alteration of fruit volatile compounds.
